# Treatment of Inflammatory Bowel Disease Associated *E. coli* with Ciprofloxacin and *E. coli* Nissle in the Streptomycin-Treated Mouse Intestine

**DOI:** 10.1371/journal.pone.0022823

**Published:** 2011-08-10

**Authors:** Andreas Munk Petersen, Susanne Schjørring, Sarah Choi Gerstrøm, Karen Angeliki Krogfelt

**Affiliations:** 1 Unit of Gastrointestinal and Serological Research, Department of Microbiological Surveillance and Research, Statens Serum Institute, Copenhagen, Denmark; 2 Department of Gastroenterology, Hvidovre University Hospital, Copenhagen, Denmark; Charité, Campus Benjamin Franklin, Germany

## Abstract

**Background:**

*E. coli* belonging to the phylogenetic group B2 are linked to Inflammatory Bowel Disease (IBD). Studies have shown that antimicrobials have some effect in the treatment of IBD, and it has been demonstrated that *E. coli* Nissle has prophylactic abilities comparable to 5-aminosalicylic acid (5-ASA) therapy in ulcerative colitis. The objective of this study was to test if ciprofloxacin and/or *E. coli* Nissle could eradicate IBD associated *E. coli* in the streptomycin-treated mouse intestine.

**Results:**

After successful colonization with the IBD associated *E. coli* strains in mice the introduction of *E. coli* Nissle did not result in eradication of either IBD associated strains or an *E. coli* from a healthy control, instead, co-colonization at high levels were obtained. Treatment of mice, precolonized with IBD associated *E. coli*, with ciprofloxacin for three days alone apparently resulted in effective eradication of tested *E. coli*. However, treatment of precolonized mice with a combination of ciprofloxacin for 3 days followed by *E. coli* Nissle surprisingly allowed one IBD associated *E. coli* to re-colonize the mouse intestine, but at a level 3 logs under *E. coli* Nissle. A prolonged treatment with ciprofloxacin for 7 days did not change this outcome.

**Conclusions:**

In the mouse model *E. coli* Nissle can not be used alone to eradicate IBD associated *E. coli*; rather, 3 days of ciprofloxacin are apparently efficient in eradicating these strains, but surprisingly, after ciprofloxacin treatment (3 or 7 days), the introduction of *E. coli* Nissle may support re-colonization with IBD associated *E. coli.*

## Introduction

The etiology of Inflammatory Bowel Disease (IBD) is so far unknown, it is, however, believed that both genetic and environmental factors are involved in the pathogenesis of IBD. Some characteristic pathological elements in IBD, such as mucosal inflammation, mucosal ulcers, and in the case of Crohn's disease, epithelioid cell granulomas also occur in infectious diseases. Therefore, many different bacteria, viruses and other microorganisms have been suspected to cause IBD. It is now well established that luminal factors in the intestine are involved in the inflammatory process of Crohn's disease (CD) and ulcerative colitis (UC). For example, diversion of the continuity of the intestines results in healing of the resting gut, whereas the inflammation will return when continuity is reestablished [1]. Furthermore, several animal models have documented the participation of intestinal bacteria in the inflammatory process [2]. More importantly, the recent finding of a defect in the caspase recruitment domain family, member 15 (NOD2/CARD15), gene among CD patients, has reawakened the search for specific pathogenic microorganisms in IBD [3]. NOD2/CARD15 is believed to be involved in the innate immune system including the production of defensins, and defects in this gene could indicate that the host is more susceptible to microorganisms [4]. It has accordingly been shown that the number of viable internalized *S. typhimurium* in Caco2 cells was higher when the Caco2 cells were transfected with a variant NOD2 expression plasmid associated with Crohn's disease [5].

*Escherichia coli* are among the most interesting bacteria in the human gut. Certain *E. coli* types with specific virulence factors are involved in childhood diarrhea, (enteropathogenic *E. coli*), tourist diarrhea (enterotoxigenic *E. coli*), and bloody diarrhea associated with hemolytic uremic syndrome (verotoxin-producing *E. coli*). Already in the 1970's, it was found that hemolytic *E. coli* were linked to active UC [6]. Furthermore, *E. coli* was linked to CD since an abundance of specific adherent-invasive *E. coli* (AIEC) was found in resected ileum from patients with CD [7], [8]. Interestingly, AIEC isolated from patients with CD have the ability to survive and replicate within the phagocytes without inducing cell death and AIEC-infected macrophages secrete large amounts of Tumor Necrosis Factor-α [9] It was shown that *E. coli* are very predominant in inflamed mucosa of patients with UC. Furthermore, these strains are “active”, based on 16 S rRNA PCR, and overrepresented in comparison with the microbiota of healthy controls, who generally had a higher biodiversity of the active microbiota [10]. Moreover, it has been demonstrated by ribosomal intergenic spacer analysis that Enterobacteriaceae are more abundant in tissue samples from patients with IBD compared to controls, and after culture, specific phylogenetic groups, B2 and D, of *E. coli* were found to be more frequent among patients with IBD [11]. Recently we showed that significantly more patients with active IBD were found to be infected with B2 *E. coli* strains with at least one positive extraintestinal pathogenic *E. coli* (ExPEC) gene compared to IBD patients with inactive disease [12]. In addition, an exuberant inflammatory response to *E. coli* has been demonstrated among patients with UC [13]. In a meta-analysis of placebo-controlled trials it was concluded that antimicrobials have some effect in the treatment of both CD [14], and ulcerative colitis [15]. Furthermore, the probiotic *E. coli* strain (*E. coli* Nissle), originally isolated during World War I from a soldier who withstood a severe outbreak of diarrhea affecting his detachment, prevents relapse of UC just as well as 5-aminosalicylic acid (5-ASA) and *E. coli* Nissle has also been proposed for maintenance therapy of Crohn's disease [16], [17]. Part of *E. coli* Nissle's probiotic abilities are presumably linked to its ability to prevent colonization of the gut with pathogenic microorganisms by producing two microcins and being able to produce a strong biofilm [18], [19]. These facts make it plausible that a combination of an antimicrobial and *E. coli* Nissle could be efficient in eradicating IBD associated *E. coli* and possibly in treating patients with IBD. The streptomycin treated mouse model was developed based on the observation that streptomycin treatment eliminated the gram-negative flora from the gut, leaving almost intact the gram-positive flora and the strict anaerobes making this model ideal for testing the ability of gram negatives to colonize the gut [20].

Our objective was, in order to understand the possible role of antimicrobials and *E. coli* Nissle in the treatment of patients with IBD, to test the ability of ciprofloxacin and/or *E. coli* Nissle to eradicate precolonized IBD associated *E. coli* strains (B2 strains isolated from patients with active IBD) in the streptomycin treated mouse intestine.

## Methods

### Ethics Statement

All animal experiments were approved by The Animal Experiments Inspectorate, The Danish Ministry of Justice (Permission no. 2007/561-1430). Animal experiments were performed by skilled personnel. Permission for collection of human specimens and recruitment of participants was obtained from the Regional Ethics Committee for Copenhagen County Hospitals (Permission no. KA03019) and all participants gave their informed written consent.

### Clinical data

The IBD *E. coli* strains were collected from patients with active ulcerative colitis (IBD1 and IBD2) and from a healthy control person, disease activity was confirmed by sigmoidoscopy, both IBD strains were of the phylogenetic group B2 and the control strain was of the phylogenetic group A. *E. coli* strains IBD1, IBD2 and the control strain were previously molecularly characterised [12] and designated as p7, p25 and c17 respectively. Both strains isolated from patients with IBD were found to be positive in five of six genes associated with extraintestinal pathogenic *E. coli* (ExPEC), while the strain from the control patient was negative for all tested ExPEC genes.

### Bacterial strains and media

All strains were tested for antimicrobial susceptibility against a panel of 18 different antimicrobials in a variety of concentrations by using Sensititre® DKMVN2. 20 ml of Muller Hilton broth (SSI nr. 1054, Copenhagen, DK) were inoculated with 10 µl of a 0.5 MacFarland (McF) solution. The inoculated Muller Hilton broth was distributed using a Sensititre AutoInoculator (TREK Diagnostic Systems, LTD, England) in the 96×microtitreplate (50 µl/well) containing the antibiotics. The microtitterplate were incubated for 18–22 h at 37°C where after the growth were visualised in Sensititre SeniTouch™ (TREK Diagnostic Systems, LTD, England) manually reading (growth/no growth) but analysed using Sensititre® Windows Software SWIN® (TREK Diagnostic Systems, LTD, England). The tested antimicrobials were amoxicillin+clavulanic acid (2∶1) 2/1–32/16 mg/L, ampicillin 1–32 mg/L, apramycin 4–32 mg/L µg/ml, cefpodoxime 0.125–4 mg/L, ceftiofur 0.5–8 mg/L, cephalothin 4–32 mg/L, chloramphenicol 2–64 mg/L, ciprofloxacin 0.03–4 mg/L, colistin 4–16 mg/L, florfenicol 2–64 mg/L, gentamicin 1–32 mg/L, nalidixic acid 4–64 mg/L, neomycin 2–32 mg/L, spectinomycin 16–256 mg/L, streptomycin 4–64 mg/L, sulphamethoxazole 64–1024 mg/L, tetracycline 2–32 mg/L and trimethoprim 4–32 mg/L. *E. coli* IBD1 and the *E. coli* control were susceptible to all antimicrobials tested. *E. coli* IBD2 strain was found resistant to ampicillin, sulphamethoxazole, streptomycin, trimethoprim and cephalothin. First a spontaneous streptomycin mutant of the *E. coli* IBD1 strain and the *E. coli* strain from a healthy control were constructed then a genetic marker transposon Tn7 kanamycin resistant gene cassette was inserted downstream of the coding region of the gene [21]. A spontaneous streptomycin and rifampicin resistant mutant of Nissle 1917, DSM 6601 was used as the probiotic strain (hereafter called Nissle). All resistant mutants were analyzed for any inhibiting/enhancing metabolic and/or growth changes and none were found. All strains were grown in Luria-Bertani (Sigma-aldrich, St. Louis, USA) media at 37°C overnight. Unless otherwise stated, the antimicrobials and chemicals used in this study were of analytical grade and obtained from Sigma-aldrich, St. Louis, USA.

### Mouse colonization experiments

Six-to-eight-week-old, outbreed albino female CFW1 mice (Harlan Laboratories, Netherlands) were used for the studies. The mice were caged in groups of three mice, and cages were changed weekly. The mice had unlimited access to food and continuously received water containing streptomycin sulphate (5 g/L) prior to inoculation with the strains and throughout the experiment. Each experiment included three mice colonized individually. Faecal samples from each mouse were tested prior to inoculation for the presence of indigenous *E. coli* with similar resistance. Inoculum suspension was prepared by overnight cultures (∼10^9^ CFU/mouse) resuspended in 20% (w/v) sucrose. Each mouse was given 100 µL bacterial suspensions orally. Faecal samples were collected individually with a 2 to 3 days interval and the numbers of CFU were determined by serially dilution and spread on selective agar plates. Selection between strain were conducted using LB-plates containing: 100 mg/L streptomycin and 25 mg/L kanamycin (for IBD1 and control strain), 100 mg/L streptomycin and 50 mg/L ampicillin (for IBD2) and 100 mg/L streptomycin and 100 mg/L rifampicin were used as selective plates for Nissle. The mice were euthanized and experimental duration time was between 3 to 6 weeks.

### Treatment of colonized mice with *E. coli* Nissle

The *E. coli* strains (IBD1, IBD2 or control) were given orally at aprox 5×10^8^ CFU/mouse once. After 6 days mice were inoculated with the probiotic strain *E. coli* Nissle given orally, aprox 8×10^9^ CFU/mouse. Colonization levels were tested daily through out the experiment by faecal cultures.

### Treatment with ciprofloxacin for 3 days

The *E. coli* strains (IBD1, IBD2 or control) were given orally at aprox 8×10^8^ CFU/mouse once. The strains were allowed to colonize the mouse intestine for 6 days before initiation of subcutaneous injections of ciprofloxacin (Ciproxin, 2 mg/ml Bayer) treatment every 6^th^ hour (0.2 mg/mouse) for 3 days. Colonization levels were tested daily through out the experiment by faecal cultures.

### Treatment of colonized mice with combinations of ciprofloxacin and *E. coli* Nissle

*E. coli* strains (IBD1, IBD2 or control) were given orally at aprox 8×10^8^ CFU/mouse. The strains were allowed to colonize the mouse intestine for 6 days before initiation of subcutaneous injections of ciprofloxacin (Ciproxin, 2 mg/ml Bayer) every 6^th^ hour (0.2 mg/mouse) for 3 day or 7 days. After antibiotic treatment mice were inoculated with the probiotic strain *E. coli* Nissle orally with aprox 9×10^9^ CFU/mouse daily. Colonization levels were tested daily throughout the experiment by faecal cultures

### Verifications of inoculated strains

Colonies on the selective plates were continuously verified as the inoculated strains by plasmid profile, PCR or Biochemical assays (MiniBactE, Statens Serum Institut, Diagnostica, Hillerød, Denmark) throughout the experiment.

Plasmid purification was described in detail by Schjørring *et al* [22]. PCR was used when verifying strains with the kanamycin resistance gene cassette insert. The primers that were used to detect the kanamycin gene cassette were: F-5′GAT GCT GGT GGC GAA GCT GT -3′, R 5′-GAT GAC GGT TTG TCA CAT GGA-3′; Original wild type (kan^s^) and the inoculated strain were used as negative/positive controls. Expand High Fidelity PCR system (2.6 U/reaction) (ROCHE Diagnostics GmbH, Mannheim, Germany) and boiling lysate were used. All PCR amplifications were performed in Peltier Thermal Cycler DNA engine DAYD™ (VWR™ international, Albertslund, Denmark) using the following PCR program: 2 min at 94°C; 30 cycles of 15 s at 94°C, 30 s at 55°C and 3 min at 72°C; and 7 min at 72°C. The PCR products were run at 50V on a 0.8% SeaKem® LE agarose gel (Lonza Rockland, ME, USA).

## Results

### Treatment of colonized mice with *E. coli* Nissle

**Figure 1:**
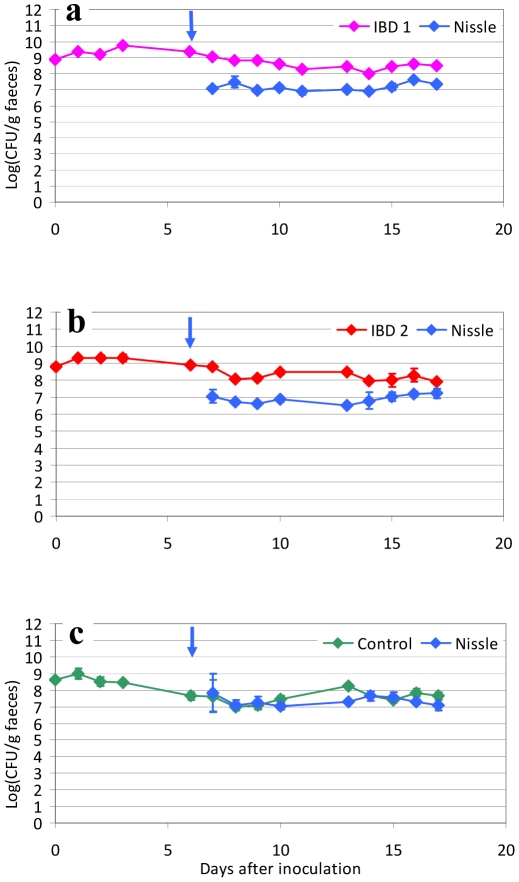
Inoculation of *E. coli* Nissle in mice pre-colonized with IBD associated *E. coli* or a control strain. Sets of three mice were used in each experiment. CFU of the inoculation suspension of IBD/control strain are shown at day 0 (∼10^9^ CFU/mouse). Blue arrow indicates the inoculation of *E. coli* Nissle strain at day 6 (∼10^9^ CFU/mouse). Each graph represents the CFU counts of three mice and bars represent Standard error of the means (SEM). Detection Limit (DL) at 20 CFU/g faeces.

To test if *E. coli* Nissle alone was efficient in eradication of IBD associated *E. coli*, mice were precolonized by two *E. coli* strains isolated from patients with active ulcerative colitis (IBD1 and IBD2) and one *E. coli* strain isolated from a healthy person. Groups of three mice were inoculated at day 0 with one of the strains IBD1, IBD2 or the control strain, and colonization was obtained at a stable level which was demonstrated by subsequent culture of faecal samples from the inoculated mice. On the sixth day after inoculation, *E. coli* Nissle was introduced by an inoculum of 10^9^ CFU given for three days. The introduction of *E. coli* Nissle did not result in eradication or any notable changes in number of CFU in samples taken from the mice until day 17 (11 days after the introduction of *E. coli* Nissle) of either IBD1, IBD2 or the *E. coli* from a healthy control; however, co-colonization was obtained (fig. 1 a–c). The two pathogenic strains IBD1 and IBD2 did show some colonization advantage compared to *E. coli* Nissle, since *E. coli* Nissle did colonize 1 log under the precolonized strain, however at a stable level (fig. 1, a and b). Mice inoculated with the commensal *E. coli* strain isolated from a healthy control co-colonized with *E. coli* Nissle and resulted in colonization at equal levels (fig. 1 c).

### Treatment of colonized mice with ciprofloxacin for 3 days

**Figure 2:**
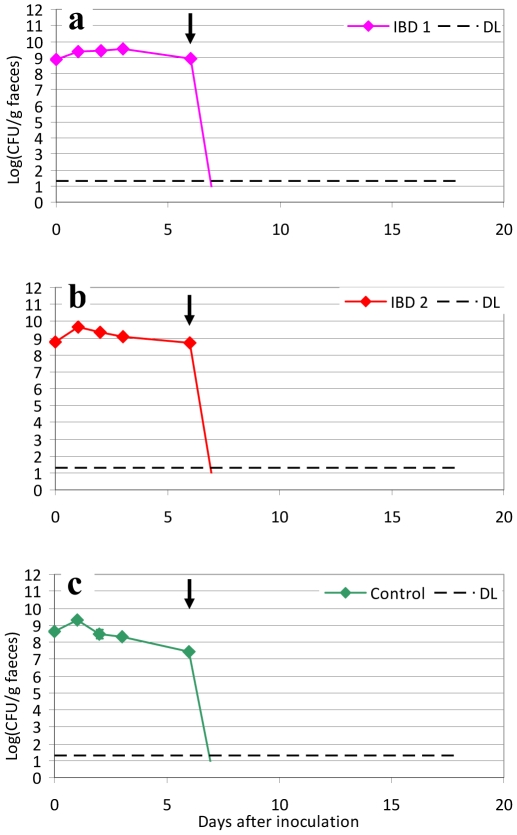
Three days treatment with ciprofloxacin in mice pre-colonized with IBD associated *E. coli* or a control strain. Sets of three mice were used in each experiment. CFU counts of the inoculated strains from faecal samples of mice. CFU of the inoculation suspension is shown at day 0 (∼10^9^ CFU/mouse). Black arrow indicates the initiation of ciprofloxacin treatment from day 6–9 (0.2 mg/mouse) every 6 h. Detection limit 20 CFU/g faeces (dotted line). Each graph represents the CFU counts of three mice and bars represent SEM. Detection Limit (DL) at 20 CFU/g faeces.

Mice were precolonized with the three test strains by inoculation of mice with 10^9^ CFU and achieving a stable colonization level. On the 6^th^ day mice were treated with subcutaneous injections of ciprofloxacin 0.2 mg/mouse every 6^th^ hour (7 am, 1 pm, 7 pm and 1 am) from day 6 to 8 after inoculation. Culture of faecal samples from the mice revealed an apparent efficient eradication of both IBD1 and IBD2, and of the strain isolated from a healthy person (control), with no *E. coli* detected for the following nine days (fig. 2 a–c), with a detection limit of 20 CFU/g faeces.

### Treatment of colonized mice with combinations of ciprofloxacin and *E. coli* Nissle

**Figure 3:**
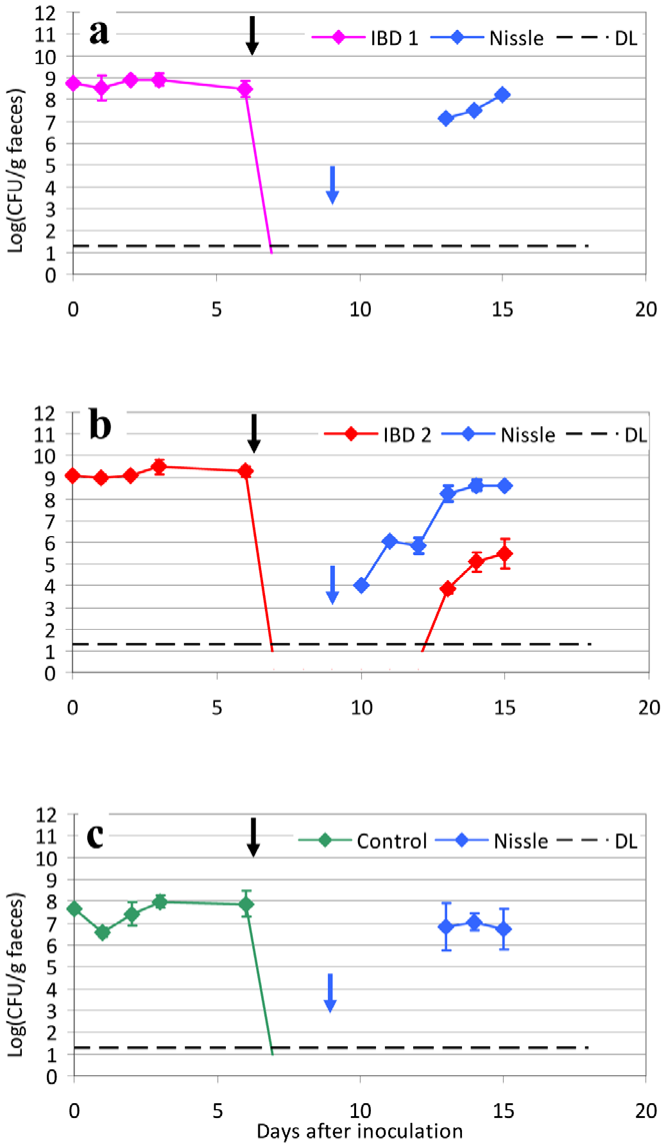
Three days treatment with ciprofloxacin and a subsequent treatment with *E. coli* Nissle daily in mice pre-colonized with IBD associated *E. coli* or a control strain. Sets of three mice were used in each experiment. CFU counts of the inoculated strains from faecal samples of mice. CFU of the inoculation suspension is shown at day 0 (∼10^9^ CFU/mouse). Black arrow indicates the initiation of ciprofloxacin treatment from day 6–9 (0.2 mg/mouse) every 6 h. Blue arrow indicates initiation of inoculation with *E. coli* Nissle strain at high levels (∼10^9^ CFU/mouse) every day throughout the experiment. Each graph represents the CFU counts of three mice and bars represent SEM. Detection Limit (DL) at 20 CFU/g faeces.

**Figure 4:**
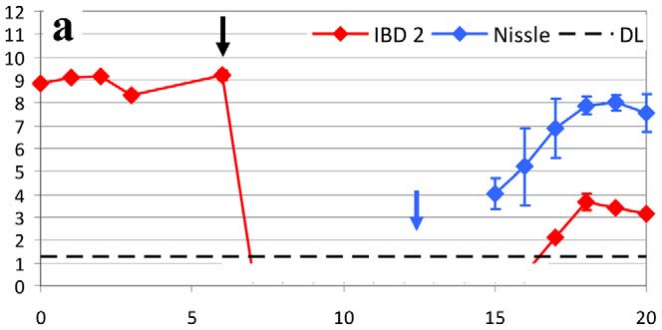
Treatment with ciprofloxacin for 7 days followed by inoculation with *E. coli* Nissle. Sets of three mice were used in each experiment. CFU counts of the inoculated strains from faecal samples of mice. CFU of the inoculation suspension is shown at day 0 (∼10^9^ CFU/mouse). Black arrow indicates the initiation of ciprofloxacin treatment from day 6–13 (0.2 mg/mouse) every 6 h. Blue arrow indicates initiation of inoculation with *E. coli* Nissle strain at high levels (∼10^9^ CFU/mouse) every day throughout the experiment. IBD associated *E. coli* strain 2 was apparently eradicated by 7 days of treatment with ciprofloxacin; however, strain 2 once again reappeared under treatment with *E. coli* Nissle. Each graph represents the CFU counts of three mice and bars represent SEM. Detection Limit (DL) at 20 CFU/g faeces (dotted line).

Since *E. coli* Nissle has previously been shown to be efficient as a prophylactic treatment in IBD, we aimed at testing the possibility of colonizing the streptomycin treated mouse intestine with *E. coli* Nissle after clearing the precolonized IBD associated *E. coli* strains from the mouse gut with 3 days of ciprofloxacin treatment. However, combination of ciprofloxacin for 3 days followed by *E. coli* Nissle for 3 days resulted in lack of colonization with *E. coli* Nissle (data not shown). Instead we chose to inoculate mice with *E. coli* Nissle 10^9^ CFU daily for the rest of the study. This is in accordance with the clinical approach in IBD patients treated with *E. coli* Nissle. With daily inoculation, *E. coli* Nissle was found in stable numbers in faecal samples from the tested mice (fig. 3 a–c). One of the tested IBD strains (IBD2) was surprisingly able to reappear after 4 days but only reaching a level 3 logs below *E. coli* Nissle (fig. 3 b). In a new set of experiments subcutaneously ciprofloxacin treatment was prolonged from three to seven days in all, however, this did not prohibit the IBD2 strain from reappearing at day 3 after the initiation of subsequent *E. coli* Nissle treatment (fig. 4). Within the study time the IBD2 strain reached a level of 4 to 5 log below *E. coli* Nissle. The reappeared IBD 2 strain was tested to see if sensitivity to ciprofloxacin was changed, and at day 19 one colony in each mouse were found to be ciprofloxacin resistant.

## Discussion

The eradication of bacteria involved in the pathogenesis of IBD and their permanent replacement with non-pathogenic, probiotic bacteria may provide a new option for the treatment of IBD and for the maintenance of remission.

The probiotic *E. coli* strain Nissle 1917 was reported to maintain remission of ulcerative colitis and pouchitis and to prevent colitis in different murine models of colitis [16], [23]–[25]. Although *E. coli* Nissle was originally isolated in 1917, the underlying mechanism of its beneficial effect in various intestinal diseases, including ulcerative colitis still remains elusive. Recently, it was shown in the streptomycin treated mouse model that *E. coli* Nissle can limit the growth of pathogenic *E. coli* O157 when administrated as treatment in precolonized mice [26]. It has been suggested that the different nutrient utilization of the different strains colonizing the intestine play an important role in the colonization ability of the strains [27]. Theoretically it is possible that *E. coli* Nissle ousts other more harmful bacteria involved in the pathogenesis of ulcerative colitis. Furthermore, *E. coli* of the phylogenetic group B2, having several virulence factors in common with ExPEC, were found in patients with active IBD. *E. coli* Nissle is also of the phylogenetic group B2 thus sharing many traits with members of this group (including uropathogenic *E. coli*) [19]. An intriguing mechanism in the prophylactic effect of Nissle in IBD could be that Nissle is able to inactivate IBD associated *E. coli* or that Nissle is able to hinder re-infection with IBD associated *E. coli*. In the present study the introduction of *E. coli* Nissle for 3 days, after precolonization of streptomycin treated mice with IBD associated *E. coli*, did not eradicate the infection with the IBD associated *E. coli*. Although *E. coli* Nissle 1917 is known to produce microcins M and H47 [18], it did not seem to be efficient in eradicating the IBD associated *E. coli*, but instead co-colonization occurred. This does not rule out that *E. coli* Nissle in the human intestine interact with the possible harmful IBD associated *E. coli* by blocking their attachment to epithelial cells. This theory could be supported by a study showing *E. coli* Nissle's ability to block the adherence of AIEC in vitro experiments with an intestinal cell line [28]. Moreover in the mouse model *E. coli* do not seem to adhere to the epithelial cells, instead *E. coli* are found situated in the mucus layer [29]. As previously mentioned, it has been demonstrated that different *E. coli* strains, including experiments with *E. coli* Nissle, can co-exist based on the utilization of different nutrients [26]. This indicates that Nissle and IBD associated *E. coli* are not competing for the same nutrients in the streptomycin treated mouse intestine. However, it was also shown that infection with three different non-pathogenic *E. coli* including *E. coli* Nissle were able to prevent recolonization with a pathogenic (enterohemorhagic) *E. coli* [26]. Therefore it is possible that *E. coli* Nissle in the human intestine, in the presence of other gram-negative bacteria, would be successful in preventing recolonization with IBD associated *E. coli*.

Ciprofloxacin for three days was efficient in eradicating the colonization of the IBD associated *E. coli*, and subsequent colonization with *E. coli* Nissle was also possible, however, only with repeated inoculation. Surprisingly, it was found that one of the IBD associated *E. coli* strains reappeared after ciprofloxacin treatment, when mice were inoculated with *E. coli* Nissle. A longer duration of ciprofloxacin treatment for 7 days did not solve this problem. Apparently in the presence of *E. coli* Nissle this IBD associated *E. coli* strain reemerged, however at lower levels than before. Several mechanisms could be speculated, first of all a subset of IBD2 *E. coli* became ciprofloxacin resistant and they could therefore survive at a dormant level. Their reawakening is perhaps effectuated by nutrients made available by *E. coli* Nissle, by crosstalk or by *E. coli* Nissle promoting the adherence of the IBD associated *E. coli* in the mucus layer, since *E. coli* Nissle has a marked ability to form biofilm [19]. It is obvious, that a combined treatment with ciprofloxacin and *E. coli* Nissle does encompass several challenges; ciprofloxacin treatment should be followed by continuous *E. coli* Nissle treatment, and the reintroduction of some IBD associated *E. coli* could be facilitated by *E. coli* Nissle. It must however be remembered that colonization is only the first step in infection and that the streptomycin-treated mouse intestine is only a model for IBD associated *E. coli* colonization and not pathogenesis. Future studies will have to elucidate the mechanisms of co-colonization, and the mechanisms by which Nissle stimulates the growth of dormant possibly ciprofloxacin resistant IBD associated *E. coli* in the streptomycin treated mouse intestine. In conclusion, the role of *E. coli* Nissle in eradication of IBD associated *E. coli* is questionable. *E. coli* Nissle alone did not eradicate any of the tested *E. coli* strains in the mouse model; instead, co-colonization occurred, and although ciprofloxacin apparently eradicated the IBD associated *E. coli,* inoculation with daily doses of *E. coli* Nissle resulted in reappearance of some IBD associated *E. coli* strains in the streptomycin treated mouse intestine.
